# Interindividual Variability Response to Resistance and High-Intensity Interval Training on Blood Pressure Reduction in Hypertensive Older Adults

**DOI:** 10.3390/jcdd12010030

**Published:** 2025-01-16

**Authors:** Johnattan Cano-Montoya, Nicolas Hurtado, Carolina Núñez Vergara, Sebastián Báez Vargas, Marcela Rojas-Vargas, Sergio Martínez-Huenchullán, Cristian Alvarez, Mikel Izquierdo

**Affiliations:** 1 School of Kinesiology, Faculty of Dentistry and Rehabilitation Sciences, Universidad San Sebastián, Valdivia 5090000, Chile; nicohurtadob123@gmail.com (N.H.); carolina.nunez@uss.cl (C.N.V.); sebastian.baez@uss.cl (S.B.V.); marcela.rojas@uss.cl (M.R.-V.); sergio.martinez@uss.cl (S.M.-H.); 2 Exercise and Rehabilitation Sciences Institute, School of Physical Therapy, Faculty of Rehabilitation Sciences, Universidad Andres Bello, Santiago 7591538, Chile; cristian.alvarez@unab.cl; 3 Navarrabiomed, Hospital Universitario de Navarra (HUN), Universidad Pública de Navarra (UPNA), IdiSNA, 31006 Pamplona, Spain; mikel.izquierdo@gmail.com; 4 CIBER of Frailty and Healthy Aging (CIBERFES), Instituto de Salud Carlos III, 28029 Madrid, Spain

**Keywords:** exercise, hypertension, inter-individual variability, older adults

## Abstract

Background: This study evaluated the effects of resistance training (RT) and high-intensity interval training (HIIT) on systolic (SBP) and diastolic blood pressure (DBP) in hypertensive older adults undergoing pharmacological therapy over four and eight weeks. We compared the efficacy of RT and HIIT in reducing non-responders (NRs) between weeks 4 and 8 and analyzed time-course adaptations in NRs and responders (Rs). Methods: Thirty-nine participants were randomized into RT-G (*n* = 13), HIIT-G (*n* = 13), or control (CG, *n* = 13) groups. RT utilized elastic bands, and HIIT involved cycle ergometers, with three weekly 30 min sessions for 8 weeks. SBP and DBP were measured before intervention and at weeks 4 and 8, respectively. Individual responses were classified as NRs or Rs using the Hopkins method (SDIR = √[SDExp2–SDCon2]). Time-course adaptations were evaluated. Results: Both the RT-G and HIIT-G reduced SBP at 8 weeks (RT-G: −13 mmHg; [ES: 1.12]; HIIT-G: −12 mmHg [ES: 0.8]; both *p* < 0.05). The proportion of NRs for SBP decreased from 46% to 38% in RT-G and 69% to 46% in HIIT-G. Rs showed a peak SBP reduction at 4 weeks (−14.7 and −25.5 mmHg), stabilizing by week 8 (−22.8 and −19.6 mmHg) in RT-G and HIIT-G, respectively. Conclusion: Eight weeks of RT and HIIT effectively reduced SBP and NR prevalence, with time-course adaptations favoring Rs.

## 1. Introduction

Hypertension (HTN) is a global public health issue and one of the leading causes of mortality worldwide [1]. Approximately 31.1% of adults globally are affected by HTN, with its prevalence increasing due to aging and greater exposure to lifestyle-related risk factors, particularly in low- and middle-income countries [2].

Physical inactivity (PI) is another significant global public health challenge [3] that is strongly associated with an elevated risk of developing primary health conditions, including HTN [4]. The prevalence and severity of this cardiovascular risk factor (CRF) increase with age [5], often necessitating greater reliance on pharmacological treatments for effective management [1].

Physical exercise has been shown to reduce blood pressure levels in hypertensive patients undergoing pharmacological therapy [6], particularly in older adults [7]. Despite the well-documented advantages of regular exercise, several barriers hinder widespread engagement. These barriers include lack of time, low motivation, relocation, health problems, and logistical challenges, such as difficulty attending exercise sessions [8,9]. To address these challenges, time-efficient exercise alternatives, such as sessions lasting no more than 30 min conducted three times per week, have been proposed as practical solutions for individuals with HTN. Resistance training (RT) [7] and high-intensity interval training (HIIT) [10] are examples of time-efficient exercise modalities that have effectively reduced blood pressure in older adults.

However, physiological adaptations to standard exercise regimens show significant inter-individual variability, with recent studies highlighting the existence of non-responders (NRs) individuals who either fail to exhibit any changes or whose favorable changes in health markers fall below the threshold of individual response in the measured variables [11,12]. Regarding systolic blood pressure (SBP), the proportion of NRs varied significantly between RT and HIIT protocols, ranging from 79% to 34% [13,14,15,16] and 75% to 53% [13,14,15,17,18], respectively. For diastolic blood pressure (DBP), NRs ranged from 76% to 54.5% for RT [13,15,19] and 72% to 54.5% for HIIT [13,15]. Despite these variations, cumulative evidence demonstrates that regular exercise produces positive effects over time [20]. Importantly, the number of NRs typically decreases after eight weeks of consistent intervention [21].

Furthermore, while most research on RT and aerobic exercise has emphasized general post-intervention outcomes, there is a growing need to explore the temporal dynamics of these adaptations (i.e., time-course changes) in blood pressure among individuals with HTN [22,23]. A better understanding of time-course adaptations could address existing knowledge gaps regarding the differential effects of exercise modalities on blood pressure over time, particularly in individuals who initially exhibit varied responses to training protocols.

Given the critical importance of evidence-based exercise prescriptions in individuals with HTN and the limited data on interindividual variability in blood pressure response over time to low-volume high-intensity exercise protocols such as RT and HIIT this study was designed to investigate the effects of these protocols on SBP and DBP in older adults with HTN. Specifically, the study aimed to evaluate the effects of four and eight weeks of two similar volumes (min/session) of low-volume, high-intensity exercise protocols (RT and HIIT) on SBP and DBP in hypertensive older adults. Additionally, this study aimed to compare the efficacy of these exercise modalities in reducing the proportion of non-responders (NRs), analyze adaptations over time in NRs and responders (Rs), and document changes in blood pressure categories [1] following the intervention.

We hypothesized that both exercise protocols would similarly reduce BP (in terms of mm/Hg) [24] and decrease the prevalence of NRs from four to eight weeks [25] for SBP and DBP reduction. Additionally, we anticipated that the reduction in SBP and DBP would be more pronounced during the initial phase of the intervention (the first four weeks) and would plateau by the end of eight weeks in Rs [22]. However, the extent of the differences in blood pressure reduction (in mmHg) between RT and HIIT protocols at four and eight weeks, as well as the variations between NRs and Rs, remain unclear.

## 2. Materials and Methods

### 2.1. Study Design

This research design corresponds to a randomized clinical trial conducted on older adults with high blood pressure. Participants were directly invited (non-probabilistic convenience sampling) during the time they were scheduled for regular health checkups. Detailed information about the research, including its associated benefits and risks, is provided. Participants of interest were asked to sign an informed consent form. Interventions (evaluations and exercise programs) were conducted between June and November 2023. The exercise program (RT and HIIT) was implemented over a period of 8 weeks, with participants completing 30 min sessions three times per week. The assessment of the variables of interest was conducted at three predetermined time points: baseline (week 0), midway through the intervention (week 4), and upon the completion of the program (week 8).

The inclusion criteria were as follows: (a) being physically inactive (not engaging in 300 or 150 min of moderate or vigorous intensity physical activity per week, respectively, as measured by the IPAQ questionnaire); (b) having a body mass index between 25 and 39.9 kg/m^2^; and (c) hypertension and enrollment in a government cardiovascular health management program. The exclusion criteria were as follows: (a) bone disease; (b) ischemic disease or arrhythmia; (c) chronic obstructive pulmonary disease (COPD) or asthma; (d) uncontrolled chronic diseases; (e) individuals unable to understand instructions; (f) individuals who did not speak Spanish; and (g) history of previous oncological disease or those under investigation for suspected neoplastic disease in any part of the body.

### 2.2. Sample Size Calculation

The sample size calculation was based on established recommendations for studies of this nature. The parameters used included: (1) ANOVA for repeated measures with within-between interaction; (2) a Type I error rate of 5%; (3) statistical power of 80%; (4) an effect size (ES) of 0.25, calculated according to the methodology reported by Lopes [26]; (5) three groups; and (6) three measurements. Based on these parameters, the recommended total sample size was 36 participants [27]. To account for a potential 30% dropout rate, the study population was increased to a total of forty-seven participants.

After the eligibility process, participants were assigned to an RT group (RT-G), an HIIT group (HIIT-G), or a control group (CG) using a 1:1:1 random allocation via an online system https://www.randomizer.org/ (accessed on 22 May 2023). The randomization sequence was generated using permuted blocks of varying sizes to ensure balanced group allocation throughout the recruitment process. Allocation concealment was performed by an investigator not involved in the clinical procedures of the study using consecutively numbered, sealed, opaque envelopes. The random sequence generation and allocation concealment method helped to control selection bias.

The RT and HIIT groups received interventions based on resistance exercise with elastic materials and high-intensity interval training (HIIT), respectively, in addition to pharmacological treatment associated with the governmental cardiovascular health management program. CG maintained its pharmacological treatment as part of the same governmental program.

**Figure 1:**
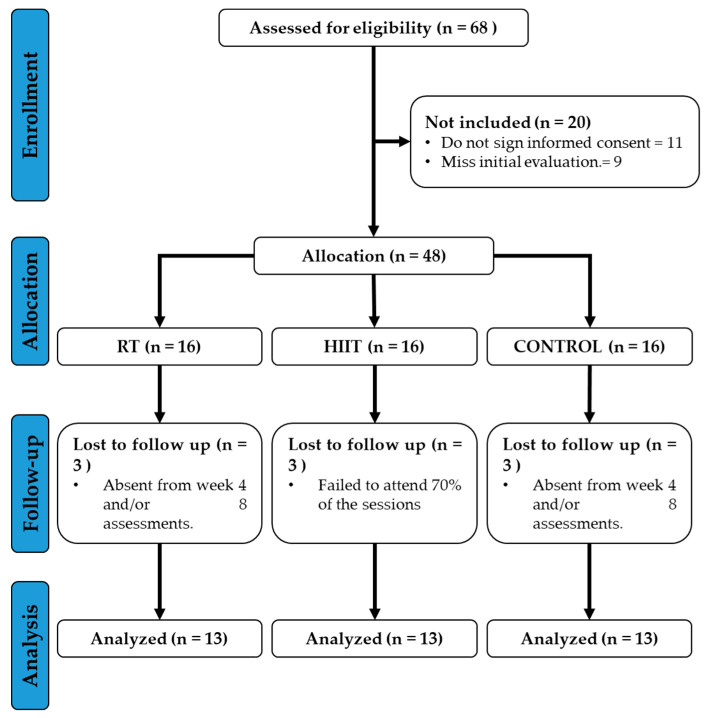
CONSORT Flow Diagram.

During the recruitment for the study, sixty-eight eligible individuals were identified during recruitment. Of these, twenty participants opted not to sign the informed consent form. The forty-eight participants who provided consent were randomly assigned to three distinct groups, with sixteen participants in each group. Throughout the follow-up period, three participants in the control group were excluded because they did not complete the scheduled evaluations at weeks 4 and 8. In the RT group, 3 participants were excluded because they did not attend at least 70% of the training sessions. In the HIIT group, another 3 participants were excluded because they did not attend the scheduled reevaluations at weeks 4 and 8. Finally, an analysis was conducted on 13 participants in each group, with the following distribution: RT (*n* = 13), HIIT (*n* = 13), and CG (*n* = 13). A flow diagram of the study participants is presented in Figure 1, following the CONSORT guidelines.

The study was conducted in accordance with the Declaration of Helsinki, adhered to the CONSORT guidelines, and approved by the Ethical Scientific Committee, Servicio de Salud Valdivia (Ord. N 166/2023). This study was a part of a trial registered at ClinicalTrials.gov (ID: NCT06201273). Date: 11 January 2024.

### 2.3. Exercise Protocols

#### 2.3.1. Resistance Training

The RT consisted of three weekly sessions for 8 weeks. Each session consisted of three distinct phases: (a) warm-up, (b) main exercise, and (c) cooldown. Prior to starting the exercise program, each participant completed three familiarization sessions that included: (1) introduction to elastic bands and instruction on exercise program procedures, (2) practice of correct exercise technique, and (3) trial implementation of resistance training (two–five sets of exercises) to understand the structure of each session. During warm-up and cool-down, all participants performed 5 min on a cycle ergometer at an intensity of 2 to 3 on the Modified Borg. During the main exercise phase, participants performed concentric and eccentric contractions using TheraBand CLX elastic bands for one minute at an intensity level of 8–10 on the OMNI-RES scale [28]. Each exercise was repeated three times, with a two-minute rest period between sets. The exercises included bicep curls, seated rows, and wide squats, as previously described [14], resulting in an average total duration of 30 min per session. The exercise load was adjusted every two weeks based on the participants’ physiological adaptations to maintain an intensity level of 8–10 on the OMNI-RES scale [28], while keeping the number of exercises, exercise duration, and rest periods constant. The resistance of the bands progressively increased in the following order: blue, black, gray, and gold. The participants’ heart rates and blood pressure were recorded before and after each session. This measure was crucial to ensure participant safety throughout the study. If the recorded values were outside the established safety limits (SBP > 180 mmHg and DBP > 110 mmHg) [1], the current session was immediately suspended, and training was resumed in the next session if the parameters were within the safe range for exercise.

#### 2.3.2. High Intensity Interval Training

Each HIIT consisted of three weekly sessions for 8 weeks. Each session consisted of three distinct phases: (a) warm-up, (b) main exercise, and (c) cool-down. Prior to starting the exercise program, each participant completed three familiarization sessions, which included: (1) introduction to the cycle ergometers and instruction on exercise procedures, (2) practice of correct exercise technique, and (3) trial implementation of the HIIT protocols (two–five intervals) to understand the structure of each session. During the warm-up and cool-down, all participants performed for 5 min on a cycle ergometer at an intensity of 2 to 3 on the Modified Borg Scale. Participants completed 8–10 intervals per session on a cycle ergometer at an intensity of 8–10 on the modified Borg scale (1–10 points) [29]. Each interval consisted of 1 min of cycling followed by 2 min of active rest, in line with previous protocols [30], resulting in an average total duration of 30 min per session. Every two weeks, if a participant did not reach an intensity of 8 on the modified Borg scale during the intervals, the resistance on the cycle ergometer was increased to maintain a consistent effort within the target range of 8–10 on the scale [30] while maintaining a constant number of exercises, exercise duration, and rest periods. Before and after each session, participants’ heart rates and blood pressure were recorded. This measure was crucial to ensure participant safety throughout the study. If the recorded values were outside the established safety limits (SBP > 180 mmHg and DBP > 110 mmHg) [1], the current session was immediately suspended, and training was resumed in the next session if their parameters were within the safe range for exercise.

### 2.4. Outcomes

#### 2.4.1. Body Composition

Height was measured using a stadiometer with a precision of 0.1 cm (Seca Bodymeter 206) [31]. The participants assumed a barefoot, upright stance, aligned their back, and heeled with the device. The participant’s head was maintained in a neutral position, facing forward, to ensure that the line of sight remained parallel to the ground. Subsequently, the horizontal rod of the stadiometer was lowered until it made gentle contact with the participant’s cranial apex and the measurement was duly recorded. Weight, percentage of body fat, and lean mass were measured using a bioimpedance analyzer (TANITA BC-534) [32]. The participants were instructed to abstain from heavy meals and intense exercise before the measurement, ensuring that they were adequately hydrated and had emptied their bladders. After removing their shoes and metallic objects, they stepped onto the scale and input their data, such as age, gender, and height [32].

#### 2.4.2. Cardiovascular Parameters

SBP and DBP were measured using an automatic monitor (Omron HEM 7130™; Omron Healthcare Inc., Lake Forest, IL, USA) [33]. To ensure accurate blood pressure measurements, participants were instructed to relax by sitting in a chair with their feet flat on the floor and their backs supported for more than five min. They were advised to avoid caffeine, exercise, and smoking for at least 30 min prior to the measurement and to empty their bladders. Neither the participants nor observers engaged in conversations during the rest period or measurement. All clothing covering the cuff placement area was removed. The cuff was positioned on the participant’s upper arm, with its middle aligned at the level of the right atrium (midpoint of the sternum) [1]. All BP measurements were taken between 8:00 and 12:00.

#### 2.4.3. Individual Responses to Exercise: NRs and Rs

Individual responses (IRs) were calculated using the equation proposed by Hopkins (43), which is given by the square root of the difference between the squares of the standard deviations of the change values in the experimental (SD_Exp_) and control (SD_Con_) groups: SD_IR_ = √(SD_Exp_^2^ − SD_Con_^2^) [34]. It should be considered that this standard deviation is the extent to which the net average treatment effect typically differs between individuals [34]. Participants were classified as NRs if they did not exhibit changes or if the changes in health markers were favorable but below the IR in the measured variables. Conversely, participants were classified as Rs if they exhibited favorable changes in health markers above IR in the measured variables. The IR was determined between the initial evaluations and those conducted at weeks 4 and 8 (pre-evaluation vs. week 4 and week 8, respectively).

#### 2.4.4. Time-Based Adaptations Between NRs and Rs

Time-based adaptations were calculated using the differential change (Δ) in SBP and DBP from baseline measurements to those recorded at the end of the fourth and eighth weeks following the approach used in previous studies [22]. This evaluation was conducted separately for individuals classified as NRs and Rs.

#### 2.4.5. Classification Blood Pressure

Before and after eight weeks of intervention, BP was categorized into four levels according to the classification by Whelton et al. [35]: normal (<120 mmHg and <80 mmHg), elevated (120–129 mmHg and <80 mmHg), HTN Stage 1 (130–139 mmHg or 80–89 mmHg), and HTN Stage 2 (≥140 mmHg or ≥90 mmHg).

### 2.5. Statistical Analyses

The dependent variables were described using the mean and standard deviation. To ensure the validity of the analyses, we verified the assumptions of normality and homoscedasticity for all data using the Shapiro–Wilk and Levene tests. To identify baseline differences in dependent variables between groups, parametric tests were used for normally distributed values and non-parametric tests were used for non-normally distributed values. Specifically, a one-way ANOVA with Tukey’s post hoc test was employed for normal distributions, whereas the Kruskal–Wallis test with Dunn’s post hoc test was used for non-normal distributions. For the analysis of both intragroup and intergroup differences over time, a two-way repeated measures analysis of variance (ANOVA) was used for normally distributed values, incorporating the model effects of Group (RT, HIIT, and Control), Time (Pre-test, Post week 4, and Post week 8), and their interaction (Group × Time). Tukey’s post hoc test was used to identify specific differences between the groups and times. For non-normally distributed values, the Friedman test replaced the two-way repeated-measures ANOVA, with Dunn’s post hoc test used to determine specific differences between groups and times. The clinical significance of the interventions was determined by effect size using Cohen’s d (<0.2 = negligible; 0.2–0.49, small; 0.5–0.79, moderate; ≥0.8, large) for interactions that showed statistical significance [36]. The IR for each variable was calculated using the equation proposed by Hopkins et al. [34], given by the square root of the difference between the squared standard deviations of the change values in the experimental (SD_Exp_) and control (SD_Con_) groups: SD_IR_ = √(SD_Exp_^2^ − SD_Con_^2^) [34]. The prevalence of participants classified as NRs or Rs was described as a percentage within the RT-G, HIIT-G, and CG groups. To evaluate the temporal dynamics of adaptation, the differential variation in delta (Δ) between baseline measurements and those recorded at the fourth and eighth week was calculated for the NRs and Rs in each group, respectively. To describe the changes in blood pressure categories [35] after the intervention, the variation in the number and percentage of participants in each category was calculated between the baseline classification and the eighth week. To ensure optimal control of bias, data analysis was conducted by a researcher who was blinded to the group assignments. All statistical analyses were performed using SPSS software version 26 (SPSS Inc., Chicago, IL, USA).

## 3. Results

### 3.1. Baseline Measurements

At baseline, there were no differences in anthropometric variables, body composition, SBP, or DBP between the groups (Table 1). Drug use and distribution according to cardiometabolic risk factors are shown in Table 1.

### 3.2. Changes in Blood Pressure According to Group and Evaluation Time

Intragroup analysis showed clinically relevant differences in the RT-G, with moderate effect sizes at four weeks (d = 0.58) and significant and clinically relevant differences at eight weeks (*p* = 0.015; d = 1.12) (Table 2). Significant differences were observed in HIIT-G at eight weeks, with a large effect size (*p* = 0.0005; d = 0.8) (Table 2). Additionally, the analysis indicated a significant effect of time on this outcome measure (F = 7.562, *p* = 0.001) (Table 2).

Regarding DBP, no significant differences were observed between the groups at the different time points, nor were there any significant intragroup differences at four and eight weeks (Table 2). Additionally, no significant effects of group (F = 0.722, *p* = 0.493), time (F = 1.803, *p* = 0.172), or group × time (F = 0.108, *p* = 0.979) were observed (Table 2).

### 3.3. Interindividual Response to Exercise: Variations in NRs

The SD_IR_ presented in this study was organized by group, outcome measure, and measurement time (weeks four and eight) as follows: for the RT-G, the IRs for PAS (mm/Hg) were −5 and −6, and for PAD (mm/Hg), −2, respectively. For HIIT-G, the IRs for PAS (mm/Hg) were −13 and −11, and for PAD (mm/Hg), −8 and −2, respectively. For the control group, the IR values from the RT-G were used, as they were the lowest among both groups for identifying the Rs for both PAS and PAD.

The prevalence of non-responders (NRs) varied across groups over time (Table 3). In the RT-G, both the PAS and PAD groups showed decreases in NRs, with reductions from 46% and 62% at week four to 38% and 46% at week eight, respectively. Similarly, HIIT-G patients experienced decreases in PAS (69% to 46%) and PAD (69% to 38%). In contrast, the CG showed increases in NRs for both PAS and PAD, from 69% to 77% and 46% to 77%, respectively (Table 3).

### 3.4. Key Findings on Time-Based Adaptations Between NRs and Rs

Time-based adaptations between NRs and Rs varied across groups (Table 3). In the RT-G, NRs exhibited increases in both PAS (2.5 to 3.4 mmHg) and PAD (2.6 to 7 mmHg), while Rs showed reductions in PAS (−14.7 to −22.8 mmHg) and PAD (−8.4 to −8.9 mmHg). In the HIIT-G, NRs showed a decrease in PAS (1.6 to −4.3 mmHg) but an increase in PAD (0.9 to 6.8 mmHg), whereas Rs experienced initial reductions in PAS (−25.5 to −19.6 mmHg) and PAD (−14 to −8.8 mmHg). In the CG, NRs demonstrated slight decreases in PAS (7.3 to 6.6 mmHg) and PAD (3.3 to 2.2 mmHg), while Rs showed consistent declines in PAS (−13 to −21.7 mmHg) and PAD (−6.6 to −16.3 mmHg).

### 3.5. Observed Blood Pressure Category Distribution After Intervention

In the RT-G (Table 4), the Normal BP category increased from 8% to 31%, while Elevated BP and HTN 1 remained stable at 31%, and HTN 2 decreased from 31% to 8%. This suggests that RT could improve severe hypertension (HTN 2) and normalize the BP in some participants. In the HIIT-G (Table 4), the Normal BP category increased from 8% to 23%, Elevated BP increased from 8% to 31%, HTN 1 decreased from 38% to 23%, and HTN 2 decreased from 46% to 23%. This indicates that HIIT was beneficial in terms of reducing both HTN 1 and HTN 2, thereby shifting participants towards a more normal BP range. In the CG (Table 4), the Normal BP category decreased from 23% to 8%, Elevated BP increased from 8% to 31%, HTN 1 decreased from 23% to 15%, and HTN 2 remained stable at 46%.

Detailed changes in the BP categories between NRs and Rs are presented in Table 5.

In the RT-G, among the five NRs, two maintained their initial conditions, and three worsened. In contrast, eight Rs for SBP improved their clinical conditions: five normalized BP values, whereas others showed shifts to lower BP categories. In the HIIT-G, all NRs maintained their initial classification, while four Rs normalized BP values, and others improved their BP categories. For the CG, five of the ten NRs experienced worsening BP, and the remaining five maintained their condition, whereas three Rs showed improvements, with two achieving normalization.

## 4. Discussion

This study aimed to evaluate the effects of two low-volume (30 min/session) exercise protocols, RT and HIIT, on SBP and DBP in hypertensive older adults over four and eight weeks. Additionally, the study sought to compare the efficacy of RT and HIIT in terms of reducing the proportion of NRs within this population, analyze the time course of blood pressure adaptations in NRs and Rs, and document changes in blood pressure categories following the intervention.

The key findings indicated that: (i) RT and HIIT protocols significantly improved SBP among hypertensive older adults. The RT-G achieved an average SBP reduction of −13 mmHg (ES: 1.12), while the HIIT-G achieved a reduction of −12 mmHg (ES: 0.8) (Table 2). (ii) Significant variability in response to exercise was observed, with a notable reduction in the number of NRs for both SBP and DBP in the RT-G and HIIT-G by the end of the intervention. (iii) The rate of blood pressure adaptation peaked during the fourth week and stabilized by the eighth week among Rs in both RT-G and HIIT-G. Finally, (iv) both RT-G and HIIT-G exhibited improvements in blood pressure classifications, highlighting the clinical relevance of these exercise protocols in managing hypertension.

### 4.1. Aggregate Effects of RT and HIIT

Physical exercise is a well-established non-pharmacological treatment for HTN [37] with effects comparable to those of antihypertensive medications [38]. High-intensity interval training has been shown to effectively reduce SBP in older adults with HTN [6]. For example, 16-week protocols of HIIT (20 min per session) have been shown to decrease SBP by −8 mmHg in hypertensive patients [39]. In our study, the HIIT group (HIIT-G) achieved a reduction of −13 mmHg within just eight weeks, demonstrating comparable results in half the time.

For RT, average SBP reductions of approximately −4.85 mmHg have been reported [37]. Studies focusing on RT protocols lasting less than 12 weeks have documented similar decreases of approximately −4.78 mmHg [7]. However, our study observed a more pronounced reduction of −12 mmHg in the RT-G over a comparable timeframe.

In contrast, significant changes were observed in the average DBP values for either protocol. This finding aligns with some studies on older adults with HTN, which have reported no DBP changes following aerobic or RT programs [40]. Nonetheless, other studies have documented modest reductions in DBP after HIIT (−2.5 mmHg) and RT (−3 mmHg) interventions in hypertensive adults [6].

The superior reductions in SBP observed in our study can likely be attributed to the high intensity and close monitoring implemented in both protocols. The HIIT protocol elicited perceived exertion values ranging from 8 to 10 on the Borg scale, while the RT protocol utilized an intensity of 8 to 10 on the OMNI-RES scale for each exercise. These intensity levels are essential for driving physiological adaptations [7,41,42].

High-intensity exercise has been shown to exert superior effects on blood pressure regulation mechanisms compared with moderate-intensity exercise in hypertensive individuals [43]. Specifically, high-intensity protocols improve endothelial function, enhance vasodilation, modulate sympathetic activity, and reduce vasoconstrictive agents such as endothelin-1, thereby lowering peripheral vascular resistance [43,44,45]. These protocols also stimulate capillary growth via vascular endothelial growth factor (VEGF), leading to increased capillary density and improved circulation [45,46].

Additional benefits of high-intensity exercise include a favorable balance between prostacyclin and thromboxane, reduced immune cell infiltration, and enhanced secretion of adiponectin, which collectively reduce inflammation and optimize vascular health [45].

High-intensity exercise emerges as an effective strategy for managing hypertension, offering both short-term (via post-exercise hypotension) and chronic improvements in vascular structure and function [43,47]. This highlights the critical role of exercise intensity in optimizing cardiovascular health benefits for individuals with hypertension.

Another potential factor contributing to the reduction in SBP is interaction with pharmacological therapy. Evidence suggests that combining exercise with antihypertensive medication may have a synergistic effect, further enhancing SBP reduction in this population [48]. Additionally, the baseline SBP level before the intervention played a role. Studies have shown that individuals with higher initial systolic blood pressure tend to experience greater reductions following exercise interventions [46]. These findings indicate that low-volume, high-intensity exercise protocols, such as HIIT and RT, represent time-efficient strategies for significantly reducing average SBP—but not DBP—in individuals with hypertension [7,41].

### 4.2. Inter-Individual Variability in Response to Exercise

Substantial variability in individual responses to exercise was observed during the initial stages of the intervention, with a notable reduction in the number of NRs after completing the 8-week program. Both SBP and DBP saw decreases in NRs, with similar trends across the two exercise protocols. Specifically, the proportion of NRs decreased from approximately 62% to 40% for SBP and from 66% to 42% for DBP, irrespective of the type of exercise performed. When comparing our results with prior studies, it becomes evident that the variability in exercise response is highly heterogeneous. For interventions lasting 12 to 16 weeks, the proportion of NRs in terms of SBP ranges from 79% to 34% in RT protocols [13,14,15,16] and from 75% to 53% in HIIT protocols [13,14,15,17,18]. For DBP, NRs ranged from 76% to 54.5% in RT [13,15,19] and from 72% to 54.5% in HIIT [13,15]. Remarkably, the present study achieved a greater reduction in the number of NRs than those described in the literature within a shorter intervention period.

Variability in exercise response is a well-documented challenge in studies assessing the effectiveness of exercise interventions. This variability is often attributed to inconsistencies in exercise dose, intensity, and training volume [21,49]. To address these issues, the present study standardized intensity and training volume across participants, with all sessions lasting 30 min, totaling twenty-four sessions. High intensity was ensured using the Borg scale (8–10) for HIIT [29] and the OMNI-RES scale (8–10) for RT [28].

Several factors may explain the reduction in non-responders (NRs) observed in this study. High adherence (>90% attendance) ensured consistent exposure to the training stimulus, which is a key element associated with a higher proportion of responders [50]. Standardized intensity and volume provided a uniform training stimulus with sufficiently potent intensities and an adequate intervention duration to elicit physiological benefits and minimize NRs [50]. Additionally, individuals with elevated baseline blood BP showed more pronounced responses, consistent with prior findings that greater initial BP levels predict greater benefits from exercise [6,18]. These results suggest that the standardization of both the protocol and the baseline BP levels contributed to the observed reduction in NRs (Table 5).

From a clinical perspective, these findings underscore the importance of adherence and protocol standardization in exercise prescription. Structured programs with controlled intensity and volume can maximize physiological adaptations and minimize NRs [50], particularly in populations with elevated baseline blood pressure. This suggests that exercise prescription should prioritize individual assessments, including baseline blood pressure, to design more effective interventions. Such an approach could enhance health outcomes by ensuring that interventions are both specific and sufficiently intense to produce measurable benefits. These insights are essential for developing evidence-based exercise guidelines aimed at reducing cardiovascular risks.

While the number of non-responders (NRs) decreased following both exercise protocols, a considerable proportion showed no change in their response profiles after eight weeks of low-volume, high-intensity exercise. This lack of response may be partially explained by baseline blood pressure (BP) values in the normal or near-normal range, where a hypotensive effect is less likely to occur, as reported previously [51]. For participants with elevated BP who remained NRs (Table 5), early identification and assessment of other factors influencing variability, such as genetics, ethnicity, age, or energy intake [52], are crucial.

The physiological mechanisms that could explain the differences in exercise response between NRs and Rs include the effects of unhealthy lifestyle factors prior to the intervention, which lead to significant systemic alterations [4]. At the vascular level, a reduction in blood flow and shear stress results in endothelial dysfunction; at the autonomic level, increased sympathetic nervous system activity induces vasoconstriction, decreased glomerular filtration rate, and increased renin release; and at the metabolic level, decreased muscle activity, muscle atrophy, ectopic lipid deposition, and insulin resistance are observed [53]. These alterations interact to generate oxidative stress; chronic low-grade inflammation; and disrupted metabolic signaling in muscles, vasculature, and blood, ultimately contributing to the development of hypertension [53]. Although exercise has been shown to reduce blood pressure through increased nitric oxide availability, improved shear stress, reduced sympathetic activity, and enhanced muscular function [54], these favorable responses may be limited by pre-existing physiological dysfunctions [55]. Furthermore, genetic variations could significantly influence the response to exercise, modulating the magnitude of its effects and amplifying the impact of unhealthy lifestyle factors on the ability to adapt to exercise [52].

Adjustments to the exercise dose, including increased volume or duration, or the incorporation of alternative pharmacological or non-pharmacological interventions may be necessary to achieve BP reduction [56].

The percentage of NRs in the CG increased from 69% to 77% for SBP and from 46% to 77% for DBP. This suggests that the absence of physical exercise or the continuation of a sedentary lifestyle, as reported by these participants, may contribute to the worsening of blood pressure levels, which is consistent with observations from previous studies [4].

### 4.3. Insights on Time-Based Adaptations Between NRs and Rs

The minimal BP reduction observed in NRs during physical exercise may be linked to environmental factors associated with allostatic load [57]. Allostatic load represents the physiological cost of chronic or excessive activation of regulatory systems, such as the nervous, endocrine, and immune systems, and is often driven by factors such as chronic stress, poor sleep quality, inadequate diet, and exposure to environmental pollutants [57,58]. This state induces oxidative stress and chronic inflammation, contributing to endothelial damage, vascular dysfunction, and the persistence of hypertension [57].

Prolonged exposure to allostatic loads may impair the body’s adaptive capacity, reducing the benefits of exercise by disrupting vascular and metabolic responses [58]. Understanding the role of allostatic load in BP regulation is crucial for the development of more comprehensive strategies for hypertension management. Future research should explore the combined impact of chronic stress, oxidative stress, inflammation, and environmental conditions to enhance the effectiveness of exercise interventions and to support long-term vascular health.

The substantial reductions in SBP and DBP observed at week 4 among responders Rs in both the HIIT-G and RT-G stand in contrast to the findings reported by Collier et al. [59]. In their study, following 4 weeks of aerobic and resistance exercise, SBP decreased by −4.5 mmHg and DBP by −3.5 mmHg [59], values which are considerably lower than those achieved by the Rs in the present study.

By week eight, BP reduction stabilized in both exercise groups, which is consistent with previous findings. Differentiated 8-week aerobic and RT protocols have shown comparable BP reductions, with SBP decreasing by −4.6 mmHg [24]. However, the reduction observed in this study was greater. Notably, previous studies did not distinguish between Rs and NRs, potentially affecting the interpretation of the outcomes. The proportional BP reductions observed with both protocols may be partially attributed to improvements in endothelial function, as reported by Pedralli et al. [24]. Mechanisms associated with enhanced endothelial function and vascular health, triggered by both HIIT [45] and RT [60], likely contribute to the effective regulation of BP.

Interindividual genetic variations may partly explain why some individuals experience greater reductions in blood pressure (BP) following exercise [61]. Physical exercise induces systemic reprogramming by activating genes involved in the regulation of metabolic and physiological changes [52]. The effects of genetic polymorphisms, which are minimal under sedentary conditions, are amplified by exercise, resulting in variability in individual responses [52]. Studies have suggested that specific polymorphisms influence gene expression post-exercise, modulating the effectiveness of training based on genotype [62]. Rs may benefit from any type of exercise due to genetic predisposition to favorable adaptations.

### 4.4. Insights on Blood Pressure Category Distribution After Intervention

Rs in the intervention groups demonstrated significant improvements in BP profiles, with more pronounced changes observed in the HIIT-G group [63]. These findings confirm the effectiveness of HIIT in producing clinically meaningful reductions in hypertension, including transitions to healthier BP categories and, in some cases, normalization, which is consistent with previous studies [48]. The combination of exercise and pharmacological therapy may further enhance the antihypertensive effects compared to pharmacological treatment alone. However, careful pharmacological monitoring is necessary to prevent hypotensive episodes in individuals who achieve normotension during long-term intervention.

The stagnation or worsening of BP categories among NRs underscores the need for personalized adjustments to exercise protocols for this population while maintaining a focus on non-pharmacological strategies, which are fundamental in hypertension management [56]. In the control group, minimal improvement was observed among Rs, whereas NRs showed significant worsening, highlighting the critical role of structured physical exercise in achieving effective and long-term BP control [56].

The observed changes in BP categories reflect not only absolute reductions in BP but also clinically significant outcomes such as reduced pharmacological dependency, lower cardiovascular risk, and decreased mortality rates [64]. In this study, reductions exceeding the minimum clinically important difference (MCID) of −5 mmHg SBP were achieved by eight participants in the RT-G and 11 in the HIIT-G, contributing to a significant decrease in HTN and cardiovascular disease risk [65]. Furthermore, modest reductions of as little as 2 mmHg in SBP or DBP, associated with a 10% reduction in stroke risk and a 7% reduction in myocardial infarction risk [66], were observed in 9 participants in the RT-G and 12 in the HIIT-G. These findings suggest that an 8-week intervention, regardless of exercise modality, effectively reduces cardiovascular risk in most participants (Table 5).

### 4.5. Strengths and Limitations

The strengths of this study are as follows: (1) its consideration of individual variability in exercise response, an essential aspect for personalizing training programs; (2) its innovative approach to documenting the time-based adaptations between Rs and NRs, which goes beyond the traditional reporting of overall intervention effects; and (3) its significant practical implications, as the findings offer valuable insights for designing physical training programs aimed at reducing blood pressure in hypertensive individuals.

The limitations of this study are as follows: (1) the limited sample size and geographic specificity, which may limit the generalizability of the results to broader or more diverse populations; (2) the relatively short duration of the study, which lasted only eight weeks—longer intervention periods could provide insights into the sustainability of improvements and reveal additional long-term changes in physical capacities; (3) the lack of control over key factors such as diet, sleep quality, chronic psychological stress, exposure to environmental pollutants, and other lifestyle variables beyond the initial recommendations provided to participants; (4) the limitations associated with using the Hopkins equation [34] to determine individual responses, which is based on the assumption that the combined effect of random variation and within-participant variation is equal between the intervention and control groups—even with random assignment to the control and intervention groups, the inability to calculate within-participant variation in each group raises the possibility that its influence may differ; (5) the potential limitation of using elastic bands for exercise intensity standardization lies in the fact that the tension generated may vary depending on the execution technique, range of motion, and positioning of the band by each individual, but detailed instructions were provided to participants regarding the correct exercise technique, proper handling of the elastic band, and appropriate intensity dosage to minimize these potential discrepancies; and (6) the gender composition of the sample, with 85% of participants being women, represents another limitation. While this predominance resulted naturally from the recruitment process and was not intentionally directed toward a specific gender, it may limit the generalizability of the findings, particularly to mixed-gender or male populations. Future studies should aim for a more balanced gender distribution to explore potential differences in responses between men and women.

Therefore, the results must be interpreted with caution to avoid undue generalizations.

## 5. Conclusions

This study demonstrates that both low-volume RT and HIIT, conducted for 30 min per session, resulted in progressive and cumulative reductions in mean SBP in hypertensive older adults undergoing anti-hypertensive therapy over an eight-week period. A reduction in the prevalence of NRs for SBP and DBP was observed between weeks four and eight. Among the Rs, the most pronounced improvements occurred during the first four weeks, followed by stabilization, achieving clinically relevant changes that improved BP profiles.

These findings highlight the efficacy of exercise interventions in delivering significant health benefits within a relatively short timeframe. Future research should focus on understanding the influence of allostatic load and genetic profiles, exploring longer intervention durations, and refining personalized exercise prescriptions. Such efforts will aim to amplify the observed effects and further decrease the proportion of NRs, enhancing the overall impact of exercise-based strategies for hypertension management.

## Figures and Tables

**Table 1:** Baseline anthropometric, body composition, and cardiometabolic risk characteristics of intervention and control groups.

Outcomes	Groups					
	RT-G Mean (SD)	HIIT-G Mean (SD)	CG Mean (SD)	RT-G vs. Control *p* Value	HIIT-G vs. Control *p* Value	RT-G vs. HIIT-G *p* Value
(***n*** = F/M)	12/1	9/4	12/1			
Age (years)	63 ± 7.02	66 ± (9.19)	66 ± (10.18)	0.696	0.996	0.641
Anthropometric						
Height (m)	1.53 ± 0.06	1.56 ± 0.09	1.53 ± 0.07	0.967	0.518	0.670
Body mass (kg)	76.02 ± 10.22	74.27 ± 13.21	73.62 ± 14.35	0.881	0.991	0.935
BMI (kg/m^2^)	32.30 ± 3.90	30.85 ± 6.53	31.57 ± 5.83	0.940	0.941	0.782
Body composition						
Body fat (%)	39.38 ± 5.83	35.46 ± 9.17	37.26 ± 7.85	0.767	0.825	0.410
Lean mass (%)	57.56 ± 5.53	61.34 ± 8.66	59.30 ± 7.90	0.825	0.768	0.412
Cardiometabolic risk						
HTN						
(***n*** = F/M)	12/1	9/4	12/1			
Drugs						
ARB (***n***)	10	7	9			
ACEI (***n***)	3	3	3			
TZD (***n***)	4	7	8			
CCB (***n***)	2	4	4			
Beta Blockers (***n***)	1	3	1			
N° Drugs						
1 (%/***n***)	62/8	46/6	31/4			
2 (%/***n***)	23/3	31/4	38/5			
3 (%/***n***)	15/2	15/2	23/3			
4 (%/***n***)	0/0	8/1	8/1			
T2D						
(***n*** = F/M)	4/0	4/2	5/0			
Drugs						
Metformin (***n***)	3	7	4			
Sulfonylureas (***n***)	0	0	1			
Insulin (***n***)	1	1	2			
N° Drugs						
1 (%/***n***)	100/4	83/5	60/3			
2 (%/***n***)	0	17/1	40/2			
Dyslipidemia						
(***n*** = F/M)	10/1	9/4	9/1			
Drugs						
Statins (***n***)	11	13	10			

Data are presented as mean ± standard deviation, (n) absolute value, and (%) percentage. Groups are described as RT-G: resistance training group; HIIT-G: high-intensity interval training group; and CG: control group. Outcomes are described as F: female; M: male, HTN: hypertension; and T2D: type 2 diabetes. Medications are described as ARB: angiotensin receptor blockers; ACEI: angiotensin-converting enzyme inhibitors; TZD: thiazide diuretics, and CCB: calcium channel blockers. Statistical effects were examined using a one-way ANOVA followed by Tukey’s post hoc test.

**Table 2:** Cardiovascular outcomes by group and time.

Outcome	Time	Groups					
		RT-G	HIIT-G	CG	Group F, (*p* value)	Time F, (*p* value)	G × T F, (*p* value)
SBP	Baseline	135 ± 13	141 ± 14	137 ± 17	1.708, (0.196)	7.562, (0.001)	1.961, (0.110)
	Wk 4	128 ± 11	134 ± 17	138 ± 16			
	Wk 8	122 ± 10 * †	129 ± 16 #	137 ± 14			
DBP	Baseline	78 ± 8	78 ± 8	81 ± 11	0.722, (0.493)	1.803, (0.172)	0.108, (0.979)
	Wk 4	77 ± 9	74 ± 9	79 ± 8			
	Wk 8	77 ± 5	75 ± 10	78 ± 10			

Data are presented as mean, standard deviation. RT-G n = 13, F/M: 12/1; HIIT-G n = 13, F/M: 9/4; CG n = 13, F/M: 12/1. Groups are described as RT-G: resistance training group; HIIT-G: high-intensity interval training group; and CG: control group. Outcomes are described as F/M: female/male; SBP: systolic blood pressure; DBP: diastolic blood pressure; and G × T, group-by-time interaction. Significant differences: * *p* = 0.015 vs. within-condition baseline; **#** *p* = 0.0005 vs. within-condition baseline, † *p* = 0.004 vs. between-condition CG. Statistical effects were examined using repeated-measures ANOVA followed by Tukey’s post hoc test.

**Table 3:** Interindividual response to exercise- and time-based adaptations between NRs and Rs.

		Groups														
Outcome	Response	RT-G					HIIT-G					C-G				
		Pre-Post Week 4		Pre-Post Week 8		w4 vs. w8 *p*-Value	Pre-Post Week 4		Pre-Post Week 8		w4 vs. w8 *p*-Value	Pre-Post Week 4		Pre-Post Week 8		w4 vs. w8 *p*-Value
		(*n* =)	Δ	(*n* =)	Δ		(*n* =)	Δ	(n =)	Δ		(n =)	Δ	(n =)	Δ	
SBP (mmHg)	NRs	6	2.5 ± 9.5	5	3.4 ± 4.5	0.409	9	1.6 ± 11.6	6	−4.3 ± 3.9	0.553	9	7.3 ± 7	10	6.6 ± 7.6	0.652
	Rs	7	−14.7 ± 9.0	8	−22.8 ± 11.8	0.132	4	−25.5 ± 11.8	7	−19.6 ± 6.6	0.447	4	−13 ± 4	3	−21.7 ± 11.4	0.629
DBP (mmHg)	NRs	8	2.6 ± 2.5	6	7 ± 7.6	0.473	9	0.9 ± 5.4	5	6.8 ± 3.4	0.07	6	3.3 ± 2	10	2.2 ± 3.6	0.473
	Rs	5	−8.4 ± 3.3	7	−8.9 ± 4.4	0.934	4	−14 ± 9.5	8	−8.8 ± 6.6	0.172	7	−6.6 ± 3.7	3	−16.3 ± 10.8	0.290

Data are presented as absolute delta (Δ) changes at weeks 4 and 8. Groups are described as RT-G: resistance training group and HIIT-G: high-intensity interval training group. Outcomes are described as SBP: systolic blood pressure and DBP: diastolic blood pressure. Exercise responses are described as NRs: non-responders and Rs: responders. (n=) indicates the number of participants. Statistical effects were examined using Mann–Whitney U test.

**Table 4:** Blood pressure category distribution among different training groups at baseline and after 8 weeks.

	Groups					
Categories of BP	RT-G		HIIT-G		CG	
	Baseline n = (%)	Week 8 n = (%)	Baseline n = (%)	Week 8 n = (%)	Baseline n = (%)	Week 8 n = (%)
Normal	1 (8)	4 (31)	1 (8)	3 (23)	3 (23)	1 (8)
Elevated	4 (31)	4 (31)	1 (8)	4 (31)	1 (8)	4 (31)
HTN 1	4 (31)	4 (31)	5 (38)	3 (23)	3 (23)	2 (15)
HTN 2	4 (31)	1 (8)	6 (46)	3 (23)	6 (46)	6 (46)

Data are presented as participant count and percentage at baseline and week 8. Groups are described as RT-G: resistance training group and HIIT-G: high-intensity interval training group. Categories of BP in adults are described as: normal (<120/<80 mm/Hg), elevated (120–129/<80 mm/Hg), HTN 1: Hypertension Stage 1 (130–139/80–89 mm/Hg), and HTN 2: Hypertension Stage 2 (≥140/≥90 mm/Hg).

**Table 5:** Blood pressure category distribution after intervention in NRs and Rs.

Groups.	Subjects	Response w8 SBP	Response w8 DBP	Baseline			Week 8			Category Transitions
				SBP	DBP	Category	SBP	DBP	Category	
**RT-G**	1	R	R	169	93	HTN 2	124	78	Normal	−3
	2	R	NR	147	73	HTN 2	116	76	Normal	−3
	3	R	R	142	81	HTN 2	119	76	Normal	−3
	4	R	NR	141	75	HTN 2	128	74	Elevated	−2
	5	R	R	132	93	HTN 2	117	82	HTN 1	−1
	6	R	R	131	81	HTN 1	121	68	Elevated	−1
	7	R	R	129	79	Elevated	110	70	Normal	−1
	8	R	R	129	78	Elevated	103	72	Normal	−1
	9	NR	NR	131	82	HTN 1	128	81	HTN 1	0
	10	NR	R	122	76	Elevated	129	73	Elevated	0
	11	NR	NR	137	65	HTN 1	145	77	HTN 2	1
	12	NR	NR	129	70	Elevated	130	83	HTN 1	1
	13	NR	NR	117	71	Normal	121	87	HTN 1	2
**HIIT-G**	1	R	R	150	78	HTN 1	120	72	Normal	−2
	2	R	R	138	81	HTN 1	116	77	Normal	−2
	3	R	R	130	74	HTN 1	119	69	Normal	−2
	4	NR	R	130	71	HTN 1	123	48	Normal	−2
	5	R	R	152	78	HTN 2	130	71	HTN 1	−1
	6	R	NR	153	74	HTN 2	130	81	HTN 1	−1
	7	R	R	138	86	HTN 1	123	74	Elevated	−1
	8	NR	NR	172	87	HTN 2	167	89	HTN 2	0
	9	NR	NR	146	78	HTN 2	141	88	HTN 2	0
	10	NR	R	145	95	HTN 2	141	84	HTN 2	0
	11	NR	NR	139	69	HTN 1	131	79	HTN 1	0
	12	NR	NR	125	70	Elevated	128	75	Elevated	0
	13	R	R	116	72	Normal	102	70	Normal	0
**CG**	1	R	R	144	88	HTN 2	113	67	Normal	−3
	2	R	R	148	90	HTN 2	123	66	Elevated	−2
	3	NR	NR	158	98	HTN 2	159	97	HTN 2	0
	4	NR	NR	158	72	HTN 2	154	80	HTN 2	0
	5	R	R	152	83	HTN 2	143	79	HTN 2	0
	6	NR	NR	146	95	HTN 2	148	96	HTN 2	0
	7	NR	NR	133	72	HTN 1	134	73	HTN 1	0
	8	NR	NR	120	70	Elevated	128	69	Elevated	0
	9	NR	NR	139	71	HTN 1	143	79	HTN 2	1
	10	NR	NR	137	88	HTN 1	150	87	HTN 2	1
	11	NR	NR	124	77	Elevated	130	80	HTN 1	1
	12	NR	NR	102	79	Normal	124	78	Elevated	1
	13	NR	NR	116	64	Normal	129	69	Elevated	1

Data are presented in mmHg at baseline and week 8, with changes shown as the delta in blood pressure category (baseline—week 8). Groups are described as RT-G: resistance training group and HIIT-G: high-intensity interval training group. Outcomes are described as SBP: systolic blood pressure and DBP: diastolic blood pressure. Exercise response are described as NRs: non-responders and Rs: responders. Categories of BP in adults are described as: normal (<120/<80 mm/Hg), elevated (120–129/<80 mm/Hg), HTN 1: Hypertension Stage 1 (130–139/80–89 mm/Hg), and HTN 2: Hypertension Stage 2 (≥140/≥90 mm/Hg). The color coding represents the transition in blood pressure categories: green indicates improvement in category (lower blood pressure), yellow indicates no change in category, and red indicates worsening (higher blood pressure).

## Data Availability

The data used in the analysis are available upon request to the corresponding author.
